# MpoxNet: dual-branch deep residual squeeze and excitation monkeypox classification network with attention mechanism

**DOI:** 10.3389/fcimb.2024.1397316

**Published:** 2024-06-07

**Authors:** Jingbo Sun, Baoxi Yuan, Zhaocheng Sun, Jiajun Zhu, Yuxin Deng, Yi Gong, Yuhe Chen

**Affiliations:** ^1^ School of Electronic Information, Xijing University, Xi’an, China; ^2^ Shaanxi Key Laboratory of Integrated and Intelligent Navigation, The 20th Research Institute of China Electronics Technology Group Corporation, Xi’an, China; ^3^ Xi’an Key Laboratory of High Precision Industrial Intelligent Vision Measurement Technology, Xijing University, Xi’an, China

**Keywords:** monkeypox, deep learning, image processing, artificial intelligence, feature selection

## Abstract

While the world struggles to recover from the devastation wrought by the widespread spread of COVID-19, monkeypox virus has emerged as a new global pandemic threat. In this paper, a high precision and lightweight classification network MpoxNet based on ConvNext is proposed to meet the need of fast and safe detection of monkeypox classification. In this method, a two-branch depth-separable convolution residual Squeeze and Excitation module is designed. This design aims to extract more feature information with two branches, and greatly reduces the number of parameters in the model by using depth-separable convolution. In addition, our method introduces a convolutional attention module to enhance the extraction of key features within the receptive field. The experimental results show that MpoxNet has achieved remarkable results in monkeypox disease classification, the accuracy rate is 95.28%, the precision rate is 96.40%, the recall rate is 93.00%, and the F1-Score is 95.80%. This is significantly better than the current mainstream classification model. It is worth noting that the FLOPS and the number of parameters of MpoxNet are only 30.68% and 31.87% of those of ConvNext-Tiny, indicating that the model has a small computational burden and model complexity while efficient performance.

## 1 Introduction

With the 2020 coronavirus pandemic having a profound impact across the globe, reports of the emergence of monkeypox in 2023 reveal the threat of another global virus (McCollum and Damon, 2014). Monkeypox (Mpox) is a disease caused by Mpox virus and is a viral zoonotic disease of orthopoxvirus, so it can be transmitted from animals to humans through direct close contact (Islam et al., 2022), as well as human-to-human transmission. Mpox was first discovered in 1958 in a monkey in a laboratory in Copenhagen, Denmark (Ladnyj et al., 1972) and is known as Mpox due to its similar outbreak symptoms to smallpox.

The Mpox virus caused the first infection in the Congo in 1970. Since then, most cases have occurred in Congo, Central and West Africa, and the number of cases has gradually increased, affecting many people living near tropical regions. As of 2022, the World Health Organization(WHO) reports that several other non-African countries such as Europe and the United States have also reported cases of Mpox virus infection (Alakunle et al., 2020).

Since the declaration of the eradication of smallpox in 1980 and the subsequent cessation of smallpox vaccination, monkeypox has emerged as the predominant orthopoxvirus. Its symptoms resemble those of smallpox, thus garnering attention in the field of public health (Mohbey et al., 2022). In 2003, the United States became the first country outside Africa to experience a monkeypox outbreak. According to reports, in September 2018, a Nigerian tourist was infected with monkeypox in Israel; in September 2018, December 2019, May 2021, and May 2022, cases of infection were also reported in Singapore; while in May 2019, July 2021, and November 2021, cases of monkeypox were recorded in the United States. These countries may be located in Southeast Asia. In May 2022, a significant number of monkeypox cases were reported in some countries where the disease does not typically occur (Mohbey et al., 2022). According to the U.S. Centers for Disease Control and Prevention (CDC), as of December 21, 2022, Mpox cases have been reported in 94 countries worldwide, totaling approximately 83,424 cases. Due to the dire impact of the COVID-19 pandemic, Mpox cases have begun to be closely monitored, showing signs of potential epidemic transmission even though large-scale transmission has not yet occurred (Rogers et al., 2008; Alakunle et al., 2020; Gürbüz and Aydin, 2022; Sharif et al., 2022). As a result, deep anxiety and worry among people are gradually spreading.

Monkeypox virus infection is generally divided into two stages: the invasive stage and the skin rash stage. Symptoms during the invasive stage include fever, severe headache, swollen lymph nodes, back pain, muscle aches, and weakness. During the skin rash stage, a rash appears 1–3 days after the onset of fever, concentrating on the face and limbs (Adalja and Inglesby, 2022). The rash progresses from macules to papules, vesicles, pustules, forms scabs, and eventually falls off. These skin lesions are typically quite painful. When the rash appears, the patient becomes contagious. Monkeypox virus can spread through contact with infected individuals or animals. Specifically, when individuals come into contact with the ulcers, scabs, respiratory droplets, or oral fluids of an infected person, it may lead to the transmission of the disease to others (Simpson et al., 2020). Therefore, timely diagnosis of this disease is essential. According to the guidelines proposed by the WHO, healthcare personnel should wear protective gear when caring for patients. Additionally, patients need to be isolated, and they should maintain distance from others. Mpox exhibits subtle differences from other viruses such as smallpox, chickenpox, and measles, primarily in the inflammatory and rash symptoms induced within the human body. Apart from polymerase chain reaction (PCR), which is an effective diagnostic method (Ibrahim et al., 2021), non-specialists may find it challenging to differentiate them visually. Moreover, PCR testing is costly and typically requires a considerable amount of time to yield results. Therefore, it has not been widely adopted, posing challenges to rapid diagnosis (Reed et al., 2004). In order to involve non-specialists in the prevention and control of Mpox virus, researchers are actively seeking effective methods utilizing artificial intelligence to identify cases of Mpox. They are engaged in data collection and research experiments to gain a deeper understanding of this disease (Banerjee et al., 2022; Khafaga et al., 2022a). The development of automated identification algorithms could not only aid in the rapid identification of Mpox virus cases but also be utilized for training healthcare professionals who are not specialized in Mpox.

Despite the relatively low fatality rate of Mpox within the range of 1–10% (Gong et al., 2022), the absence of an antiviral therapy for curing monkeypox (Reynolds et al., 2017) underscores the importance of early detection in preventing its spread. Early identification plays a crucial role in the prevention, diagnosis, and treatment of this disease. With the rapid advancement of artificial intelligence models in the field of medicine, deep learning models for medical image analysis have been proposed for various medical science applications. However, there still exist numerous traditional manual classification methods for identifying Mpox, which suffer from apparent inefficiencies and high costs. The reliance on manual diagnostic classification is susceptible to human factors, leading to diagnostic variability and delayed confirmation of Mpox, depriving patients of timely access to appropriate treatment plans. Therefore, there is an urgent need to introduce advanced technologies to replace simplistic manual classification methods, thereby enhancing the chances of patient recovery and reducing the risk of transmission.

Over the years, deep learning (DL) has achieved remarkable success, exerting a profound impact on the core concepts of machine learning (ML) and artificial intelligence (AI). DL methods have demonstrated outstanding results in various industrial domains, overcoming limitations of traditional approaches. They have become powerful tools in the fields of image analysis and pattern recognition, showing extensive potential applications in disease detection. In particular, Convolutional Neural Networks (CNNs) have emerged as a cornerstone in image recognition, owing to their exceptional capabilities in feature extraction and non-linear representation. CNN, a DL neural network architecture, is commonly employed with image data as input. Through a series of operations, it extracts crucial features and information from input images, facilitating tasks such as classification or other related objectives. Some scholars have made significant progress by applying deep learning techniques to disease classification tasks. Sandeep et al. proposed a low-complexity CNN for identifying skin diseases such as psoriasis, melanoma, lupus, and chickenpox (Sandeep et al., 2022). Their research found that using existing VGGNet and image analysis techniques could accurately detect 71% of skin diseases. However, their proposed solution demonstrated optimal performance with an accuracy of approximately 78%. Glock et al (Glock et al., 2021). utilized the ResNet-50 model to develop a transfer learning approach for measles detection, exhibiting good performance on multiple rash image datasets with a sensitivity of 81.7%, specificity of 97.1%, and accuracy of 95.2%. Velasco et al (Velasco et al., 2019) introduced an intelligent smartphone skin disease recognition method based on MobileNet, reporting an accurate detection of chickenpox symptoms with an accuracy of about 94.4%. Sahin et al. (Sahin et al., 2022) developed a mobile application on the Android platform that rapidly diagnoses Mpox patients using deep learning technology, achieving an image classification accuracy of 91.11%.

Some scholars have already applied deep learning to the task of Mpox classification, achieving significant results (Mehrotra et al., 2020; Ahsan et al., 2023; Almufareh et al., 2023). A portion of researchers has also conducted relevant studies in the field of Mpox disease identification, as elucidated in the “Literature Review.” The main objective of this study is to determine and validate the optimal performing model for Mpox classification through transfer learning methods and classification models, with the aim of determining the incidence rate of Mpox. This study introduces a Mpox classification algorithm named MpoxNet, based on ConvNext (Liu et al., 2022), designed for the task of Mpox disease classification. Improvements to the ConvNext model are as follows: firstly, the introduction of a new dual-branch deep residual Squeeze and Excitation (D^2^RSE) module replaces the original ConvNext. This module has a dual-channel structure, significantly enhancing the model’s classification accuracy while achieving better performance with a reduced number of model parameters. By integrating the Convolutional Block Attention Module (CBAM) with ConvNext, we capture the spatial relationships of Mpox features during training, adaptively weighting features based on their importance in different spatial and channel dimensions, and suppressing irrelevant regions, allowing the model to selectively focus on features of different disease categories. For example, Sitaula et al. (Sitaula and Hossain, 2021)proposed a deep learning model based on attention, which employed the attention mechanism of VGG-16, aiming to more accurately capture the spatial relationships of key regions in chest X-ray images. In this experiment, MpoxNet is compared with other mainstream algorithm models, demonstrating higher classification accuracy in Mpox classification scenarios. The main contributions of this paper are as follows:

1. The introduction of a dual-branch D^2^RSE module, along with the integration of CBAM to enhance the ConvNext model, facilitates the effective extraction of critical local and global information regions within Mpox images. As a result, the proposed MpoxNet model demonstrates a notable improvement in the accuracy of monkeypox detection.
2. Conducted comprehensive ablation experiments to independently verify the impactful roles of the D^2^RSE module and CBAM attention mechanism in the model’s performance. This contributes to a more profound understanding of Mpox disease, offering valuable insights for future research.
3. Our proposed method requires fewer parameters compared to other mainstream networks and is trained end-to-end, which adequately validates the superiority of the network in terms of performance.

This paper is organized into the following sections: The second section outlines existing methodologies in Mpox image classification. The third section provides insights into the Mpox dataset and introduces the proposed enhancements. The fourth section details the performance evaluation and experimental results. Finally, the fifth section offers a comprehensive summary of the paper.

## 2 Literature review

Skin lesions are prevalent in clinical practice, making precise detection and diagnosis crucial for accurate patient treatment. In recent years, the emergence of machine learning technologies has provided significant potential to assist in the identification and clinical decision-making for skin lesions (Fraiwan and Faouri, 2022). In this section, we will present various artificial intelligence techniques, including machine learning, CNNs, and transfer learning algorithms, applied for the detection and diagnosis of Mpox virus.

Iftikhar et al. (Iftikhar et al., 2023) proposed a novel filtering and ensemble technique for the rapid and accurate prediction of Mpox cases. This approach generates two subsequences, long-term trend sequences, and residual sequences, through filtering, and employs five machine learning models for prediction. Mandal et al. (Mandal et al., 2022) introduced a clustering method for Mpox cases that combines machine learning and Particle Swarm Optimization (PSO). Bhosale et al. (Bhosale et al., 2022) conducted similar work, utilizing linear regression, decision trees, random forests, elasticNet, and ARIMA for the prediction of Mpox cases. They further introduced a dataset named Mpox Skin Image Dataset (MSID), widely employed in various studies. For instance, Khafaga et al. (Khafaga et al., 2022b) introduced a novel framework for the classification of Mpox disease images. They employed the Random Fractal Search (BERSFS) using the Al-Biruni Earth Radius (BER) optimization method for fine-tuning on deep CNN layers. Saleh and Rabie et al. (Saleh and Rabie, 2023) proposed a Human Mpox Diagnosis (HMD) strategy based on artificial intelligence technology. This strategy comprises two key components: utilizing Improved Binary Chimpanzee Optimization (IBCO) and selecting features with significant value for transfer to the diagnostic model of ensemble learning. Ultimately, HMD achieved an accuracy of 0.98. Ahsan et al. (Ahsan et al., 2023)developed a Mpox diagnostic model using the Generalization and Regularization-based Transfer Learning Approach (GRA-TLA) for binary and multiclass classification. They tested ten different Convolutional Neural Network (CNN) models, including both binary and multiclass tasks, in three independent studies. In studies one and two, their model combined with Extreme Inception (Xception) achieved accuracies ranging from 77% to 88% in distinguishing individuals with and without Mpox. In study three, the accuracy using the ResNet-101 network ranged from 84% to 99%. Kumar et al. (Kumar, 2022) employed skin images for Mpox disease diagnosis, using various CNN models and machine learning algorithms. They extracted image features using Vgg16Net and AlexNet and applied classifiers such as Naive Bayes, Decision Trees (DT), K-Nearest Neighbors (KNN), Support Vector Machine (SVM), and Random Forest. The Naive Bayes algorithm combined with Vgg16Net achieved the highest accuracy at 91.11%.

Research on skin lesion image classification through the combination of CNN and transfer learning methods has yielded significant benefits. Ahsan et al. (Ahsan et al., 2022) conducted an initial investigation into Mpox diagnosis. The authors collected images of patients infected with Mpox from various accessible portals and proposed a VGG16-based model for Mpox diagnosis. They constructed their dataset by gathering images from Google and utilized transfer learning to create a model based on the VGG16 architecture in two separate studies. The first study successfully distinguished Mpox from chickenpox, achieving an accuracy of up to 0.97, while the second study differentiated Mpox from other diseases (chickenpox, measles, and normal skin) with an accuracy of 0.89. Meena et al. (Meena et al., 2023) proposed a hybrid technique based on Convolutional Neural Networks (CNNs) and Long Short-Term Memory networks (LSTMs) to embed knowledge graphs into various healthcare applications, providing enhanced data representation and knowledge inference. Experimental results demonstrate that the proposed model achieves an accuracy of 94% on the Mpox dataset. Bala et al. (Bala et al., 2023) presented a Convolutional Neural Network (MonkeyNet) based on an improved DenseNet-201 for Mpox image recognition. They evaluated its performance using the original images from the MSID dataset for training. Their model accurately identified Mpox on both the original and augmented datasets, achieving accuracies of 93.19% and 98.91%, respectively. Jaradat et al. (Jaradat et al., 2023) evaluated five pre-trained models, including VGG19, VGG16, ResNet50, MobileNetV2, and EfficientB3. Experimental results demonstrated that MobileNetV2 performed the best, achieving an accuracy of 0.98. Model validation across different datasets confirmed MobileNetV2’s highest accuracy of 0.94. Altun et al. (Altun et al., 2023) employed a similar approach and developed a hybrid function learning model incorporating hyperparameter optimization. They utilized custom models, including MobileNetV3-s, EfficientNetV2, ResNet50, Vgg19, DenseNet121, and Xception. Notably, the optimized MobileNetV3-s model exhibited the best performance, achieving an accuracy of 0.96. Uzun Ozsahin et al. (Uzun Ozsahin et al., 2023) applied deep learning models such as AlexNet, VGG16, and VGG19 for the detection task on Mpox and chickenpox datasets. Through their research methodology, they achieved a highest precision of 0.99. Sitaula et al. (Sitaula and Shahi, 2022) utilized deep learning techniques for Mpox diagnosis. They compared 13 pre-trained deep learning models, ultimately selecting the most outstanding model to build their system. The results indicated an accuracy of 0.87 for their Mpox diagnostic model. Ali et al. (Ali et al., 2022) created a dataset named “Monkeypox Skin Lesion Dataset (MSLD)” designed for automatic detection of Mpox disease from skin lesions. The images were primarily sourced from websites, news portals, and publicly available case reports. They employed pre-trained models such as VGG-16, ResNet50, and InceptionV3 for Mpox classification. To enhance the accuracy of Mpox detection, Sahin et al. employed six deep learning models, including ResNet-18, MobileNet, NasNetMobile, GoogLeNet, EfficientB0, and ShuffleNet, to distinguish Mpox images from images depicting other diseases. Among these models, MobileNet exhibited the best performance, achieving a 91.11% accuracy in image classification. Additionally, Almufareh et al. (Almufareh et al., 2023) utilized various independent CNN architectures to differentiate Mpox from non-Mpox cases. They validated their models using MSID and MSLD datasets. Javelle et al. (Javelle et al., 2023) redefined the emerging Mpox disease and designed a self-management questionnaire for case management, contact monitoring, and support for clinical research. Haque et al. (Haque et al., 2022) employed five deep learning models, VGG19, Xception, DenseNet121, EfficientNetB3, and MobileNetV2, and integrated spatial attention mechanisms for accurate classification of human Mpox. Yasmin et al. (Yasmin et al., 2022) obtained Mpox images from the Kaggle global dataset and used nine models for predictions. Among them, the optimal predictive model achieved an MSE value of 41922.55, R2 of 0.49, MAPE of 16.82, MAE of 146.29, and RMSE of 204.75. Meena et al. (Meena et al., 2024) established a deep learning model based on transfer learning to assist in diagnosing whether patients have Mpox. In the experiment, the InceptionV3 model they used achieved an accuracy of 98%.

**Table 1** summarizes the relevant studies on Mpox diagnosis. However, research on Mpox diagnosis using deep learning remains relatively limited. Some investigations have assessed the potential of deep learning algorithms in identifying this disease. While the results suggest that deep learning could be a valuable tool for the diagnosis and control of Mpox, further research is needed to validate these findings and establish practical applications in real clinical settings.

**Table 1:** Summary of literature review.

Methodology	Approaches	Dataset	Best Results
Sahin et al. (Sahin et al., 2022)	ResNet18, GoogleNet, EfficientNetB0, NasnetMobile, ShufeNet, and MobileNetV2	MSLD	Accuracy: 0.91
Ali et al. (Ali et al., 2022)	VGG16, ResNet50, and InceptionV3	Custom Dataset	Accuracy: 0.82
Ahsan et al. (Ahsan et al., 2022)	VGG16	Custom Dataset	AUC: 0.97
Kumar et al. (Kumar, 2022)	CNN models AlexNet, GoogleNet and VGG16Net with Naïve Bayes, SVM, KNN, Random Forest, and Decision Tree	Ali et al. (Ali et al., 2022)	Accuracy: 0.91
Jaradat et al. (Jaradat et al., 2023)	Xception, DenseNet	Custom Dataset	Accuracy: 0.98 Precision: 0.99 Recall: 0.96 F-score: 0.98
Altun et al. (Altun et al., 2023)	CNN model based on MobileNetV3-s, EfficientNetV2, ResNet50, VGG19, DenseNet121, and Xception models	Custom Dataset	Accuracy: 0.96
Sitaula et al. (Sitaula and Shahi, 2022)	VGG-16, VGG-19,ResNet50, ResNet101,IncepResNetv2, MobileNetV2, InceptionV3, Xception, EfficientNetB0, EfficientNetB1, EfficientNetB2, DenseNet121 and DenseNet169	Ahsan et al. (Ahsan et al., 2022)	Accuracy: 0.85
Yasmin et al. (Yasmin et al., 2022)	Polynomial Regression, SVR, Holt’s Linear Model AR Model, SARIMA Model ARIMA Model, MA Model, Holt-Winter’s Model, and Prophet Model	Custom Dataset	MSE: 41,922.55 R2: 0.49 MAPE: 16.82 MAE: 146.29 RMSE: 204.75
Alwakid et al. (Alwakid et al., 2022)	ResNet50	HAM10000	Accuracy: 0.86 Precision: 0.84 Recall: 0.86 F-score: 0.86
Abdelhamid et al. (Abdelhamid et al., 2022)	Binary PSOBER algorithm	Bala et al. (Bala et al., 2023)	Accuracy: 0.98
Proposed Method	ConvNext	Custom Dataset	Accuracy: 0.97 Precision: 0.96 Recall: 0.93 F-score: 0.95

## 3 Materials and proposed methods

**Figure 1:**
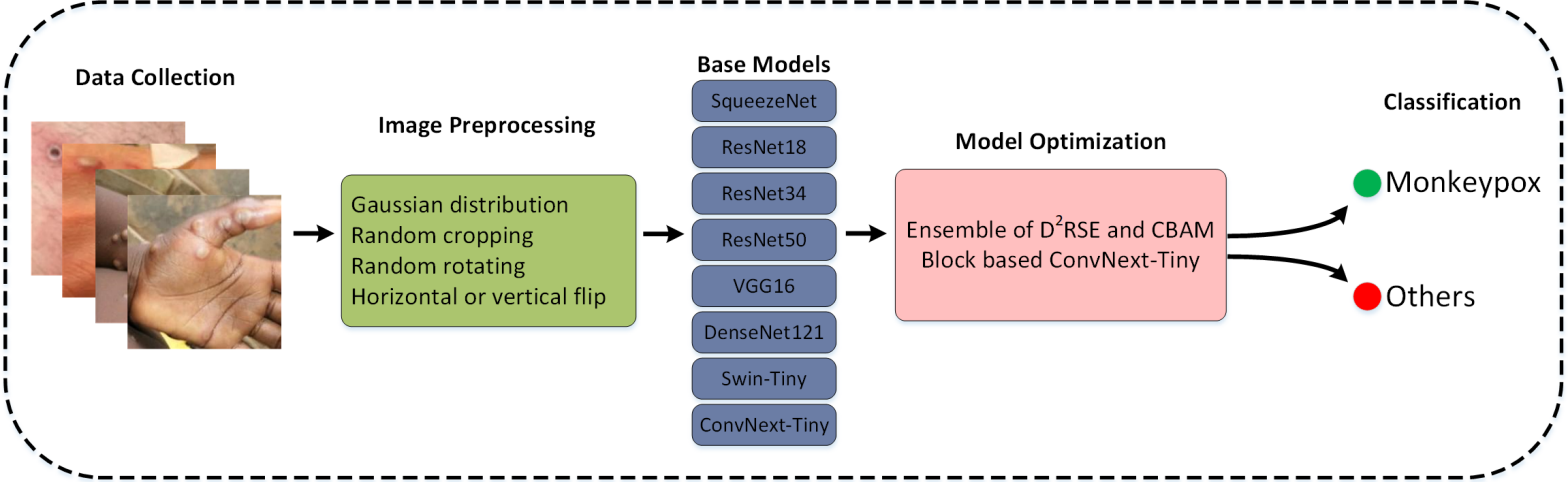
Processes diagram of the proposed method.

This section encompasses four key stages: data collection, image preprocessing, model selection, and model optimization. In the first stage, we utilized the Monkeypox Skin Image Dataset (MSID), which is freely accessible on the Kaggle platform (Monkeypox Skin Images Dataset (MSID), n.d).. And due to limited data, we employed data augmentation techniques to generate additional images. During the data preprocessing phase, the collected images underwent operations such as resizing, normalization, and data augmentation, which are crucial for enhancing model performance. We selected eight commonly used models (SqueezeNet, ResNet18, ResNet34, ResNet50, Vgg16, DenseNet121, Swin-Tiny, and ConvNext-Tiny) for comparison to improve the accuracy of the Mpox virus detection model. In the model training phase, the selected models were trained using the preprocessed images, optimizing model performance by providing images and adjusting parameters. Finally, in the evaluation stage, metrics such as accuracy, precision, recall, and F1 score were employed to assess the models, with the best-performing model selected as the final model. Therefore, this approach utilizes deep learning techniques to analyze Mpox images, accurately classifying and diagnosing the disease based on visual features. The algorithm demonstrates high accuracy in disease classification, providing a valuable tool for the rapid and precise diagnosis of Mpox in clinical settings. The proposed method comprises multiple stages and processes, illustrated in **Figure 1**, which encompass various steps and operations.

### 3.1 Data collection

This study utilized the Mpox Skin Image Dataset (MSID), a freely available resource on the Kaggle platform (Monkeypox Skin Images Dataset (MSID), n.d)., and categorized the images into two classes: Mpox and non-Mpox. This dataset serves as a crucial resource for in-depth research on the Mpox virus and its impact on human health. The dataset includes high-resolution images that intricately depict the manifestations of Mpox at different stages, as well as the infection symptoms it induces on human skin. The dataset provides detailed visual information on skin lesions, rashes caused by Mpox, and images related to other skin conditions, offering comprehensive visual insights. By encompassing a large number of images covering various aspects of Mpox, researchers are able to delve into the development of Mpox disease and identify crucial features that contribute to accurate diagnosis. Leveraging the outstanding performance of deep learning models in image enhancement, this study fully capitalized on their advantages.

### 3.2 Image preprocessing

**Figure 2:**
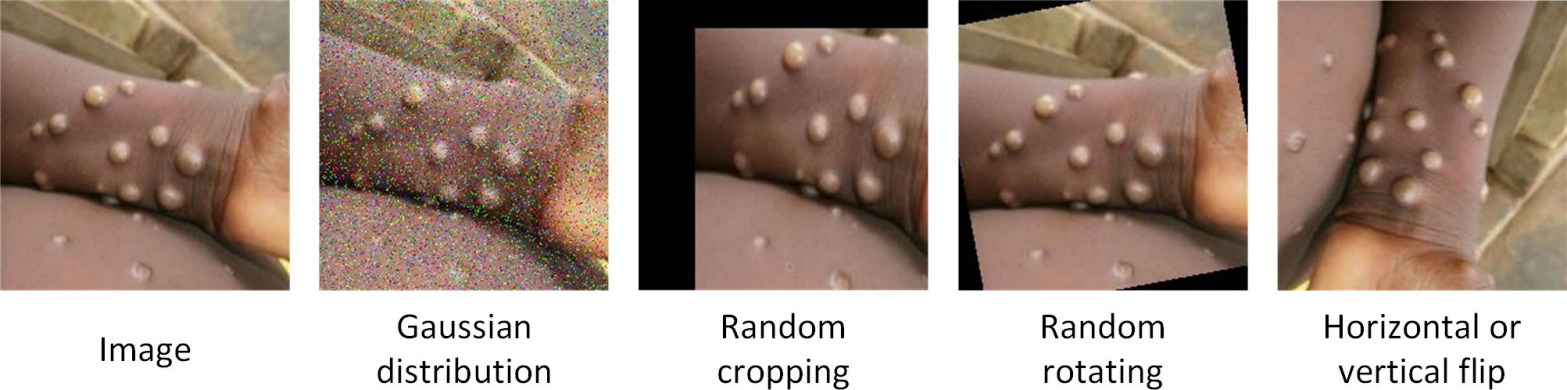
Mpox images enhanced using data augmentation.

The preprocessing stage plays a crucial role in image analysis as it contributes to enhancing data quality and consistency. The preprocessing steps in this study include image scaling and data augmentation. In this research, considering the trade-off between training speed and accuracy, images were resized to 224×224 pixels, facilitating easier handling by the model due to the uniformity of image sizes. **Figure 2** illustrates how data augmentation techniques were applied to enhance the quality of Mpox samples, thereby expanding the dataset and preventing overfitting. Through this augmented dataset, the model’s performance was successfully improved, and overfitting was mitigated. The following methods were employed for data augmentation on Mpox images:

Gaussian Noise: Adding Gaussian-distributed noise to the images simulates random signal interference during capture or transmission processes, providing a way to assess the algorithm’s performance in real-noise environments.

Random Cropping: Randomly cropping images is a common data augmentation method that increases the variation range of images, enhancing the model’s generalization capability and robustness.

Random Rotation (0–360 degrees): Randomly rotating images without altering the original image information helps reduce overfitting and improve model efficiency.

Horizontal or Vertical Flipping: Flipping images horizontally or vertically based on probability.

Finally, the Mpox dataset comprises 2000 images after augmentation, with 1400 images used for model training, 400 for testing, and 200 for validation.

### 3.3 Basic architecture selection

In order to enhance the classification performance on the augmented Mpox dataset, this study drew inspiration from algorithms used in other research [23, 29, 48]. However, the relevant literature did not disclose the code in their papers, and there were differences in the datasets used, making it challenging to directly compare the proposed algorithm with theirs. Therefore, this paper evaluated the classification performance of the base models [SqueezeNet (Iandola et al., 2016), ResNet18 (He et al., 2015), ResNet34, ResNet50, Vgg16 (Simonyan and Zisserman, 2015), DenseNet121 (Huang et al., 2018), Swin-Tiny (Liu et al., 2021), and ConvNext-Tiny (Liu et al., 2022)] employed on the Mpox dataset. The reason for selecting these models is that most of them have been widely applied in the literature. Additionally, each model has a unique architecture and strengths, and combining their strengths may contribute to improving overall performance. The experimental results on the test set are presented in **Table 2**. On the test set, ConvNext-Tiny demonstrated the best generalization performance with an accuracy of 94.33%. Therefore, building upon this foundation, the paper further improved and proposed MpoxNet, which exhibits superior performance. After our enhancements, MpoxNet is much lighter than ConvNext-Tiny, with parameters reduced by less than 68.13%, while achieving a higher accuracy of 0.95%. This makes MpoxNet more suitable for the classification diagnosis of Mpox.

**Table 2:** Results of different network on MSID dataset.

	Accuracy(%)	Precision(%)	Recall(%)	F1-score(%)	Flops(G)	Params(M)
SqueezeNet	74.52	71.60	71.10	77.10	**23.44**	**0.73**
ResNet34	81.76	76.60	85.20	82.70	117.70	21.28
ResNet50	85.53	88.10	78.20	87.50	132.21	23.51
ResNet18	88.67	87.90	86.60	89.80	58.35	11.17
VGG16	90.88	89.00	90.80	91.70	495.04	138.35
DenseNet121	91.50	90.20	90.80	92.30	92.67	6.95
Swin-Tiny	92.76	93.40	90.10	93.60	139.88	27.50
ConvNext-Tiny	94.33	92.50	91.70	94.80	142.55	27.80
MpoxNet(Our)	**95.28**	**96.40**	**93.00**	**95.80**	43.74	8.86

The best results are highlighted in bold text.

### 3.4 ConvNext

**Figure 3:**
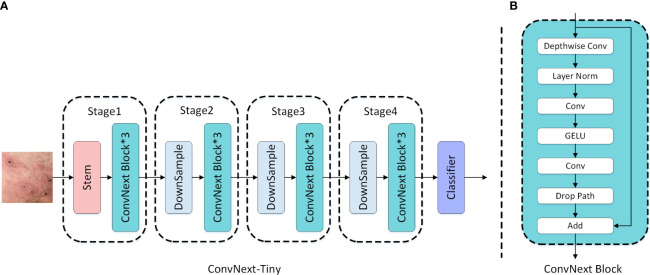
**(A)** ConvNext-Tiny network structure; **(B)** ConvNext Block structure.

ConvNext is a pure CNN model proposed by Liu et al (Liu et al., 2022), designed to eliminate cumbersome operations such as window movement and relative position deviation, providing superior performance and lower computational burden compared to popular transformer networks. The overall structure of ConvNext is based on the ResNet design, incorporating residual blocks and combining various advanced network design techniques to further enhance the overall performance of the network. The detailed structures of ConvNext-Tiny and ConvNext blocks are illustrated in **Figure 3**.

### 3.5 D^2^RSE block

**Figure 4:**
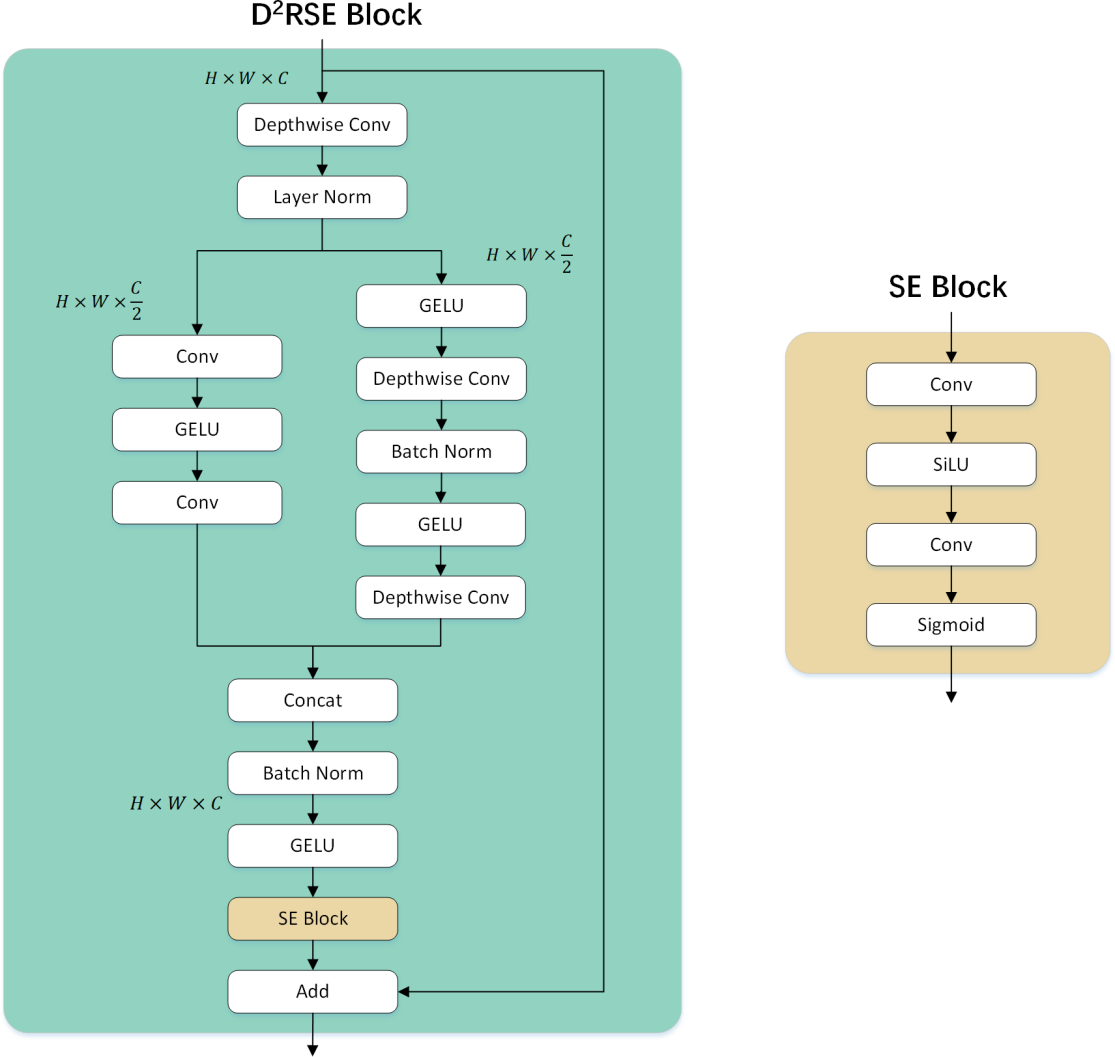
Configuration of D^2^RSE and SE Block.

In terms of improving accuracy, increasing the cardinality of the network is more effective than increasing depth or width. This viewpoint was initially proposed in ResNeXt (Xie et al., 2017; Jiang et al., 2023). Inspired by this, this paper introduces a Dual-Branch Depthwise Separable Convolution Residual Squeeze and Excitation (D^2^RSE) module in ConvNext. One branch follows a traditional modular design, while the other branch adopts the unique design of two consecutive convolutions in the Wide Residual Network. The dimensions of both branches are set to half of the main branch. Meanwhile, to reduce the number of model parameters, depthwise separable convolutions are used instead of traditional convolutions. Subsequently, by concatenating the output dimensions of the two branches and applying operations such as Batch Normalization and GELU, overfitting of the model is effectively prevented, thereby improving overall performance. Finally, an SE (Squeeze and Excitation) block is added after GELU. It explicitly models interdependencies among convolutional feature channels, allowing adaptive allocation of weights for different channels. This enables the network to perform dynamic channel-wise feature recalibration, enhancing the representation capacity of the network. Through this mechanism, the network learns to selectively emphasize informative features using global information and suppress less useful features. Furthermore, a residual connection is established between the original output and the final layer output, effectively increasing the depth of the model and enhancing its representational capacity and performance. In addition to increasing depth, the residual connection facilitates more effective learning of critical features by providing a mechanism for direct connections across layers, further improving overall performance. The D^2^RSE and SE modules are illustrated in **Figure 4**.

Specifically, the input feature map of the D^2^RSE block is processed through depthwise separable convolution and LayerNorm. The feature map captured by depthwise separable convolution and LayerNorm can be represented as shown in Equation 1:


(1) $${\text{F}}^{7}=\text{L}\left({\text{W}}_{7\times 7}\times {\text{F}}_{\text{input}}\right)$$

where $F_{input}\in {\mathbb{R}}^{c\times h\times w}$ represents the input feature map, $W_{7\times 7}$ denotes the 7×7 convolution matrix, and L(·) represents Layer-normalization. Subsequently, the feature map is integrated into each branch. To extract valuable target features from the feature map, we first design two convolutional modules to extract features, guiding the network to learn more robust feature representations. The obtained features can be shown as in Equation 2:


(2) $$F^{L}=W_{fc}\left(\sigma_{r}\left(W_{fc}\times {\text{F}}^{7}\right)\right)$$

First, we input the processed combined feature map $F^{7}\in {\mathbb{R}}^{\frac{c}{2}\times h\times w}$ into the left branch to compress it into a new feature map $F^{L}\in {\mathbb{R}}^{\frac{c}{2}\times h\times w}$ . Here, $W_{fc}$ and $\sigma_{r}\left(\cdot \right)$ represent the fully connected layer matrix and the GELU activation operation, respectively. Meanwhile, we use depthwise separable convolution instead of regular convolution to reduce the number of model parameters. Additionally, GELU activation and Batch Normalization are applied and shown in Equation 3.


(3) $$F^{R}={\text{W}}_{3\times 3}\left(\sigma_{r}\left(\text{B}\left({\text{W}}_{3\times 3}\times \sigma_{r}\left({\text{F}}^{7}\right)\right)\right)\right)$$

It is noteworthy that these two-branch maps help us extract more representative feature maps from different scales of receptive fields. The feature maps $F^{L}\in {\mathbb{R}}^{\frac{c}{2}\times h\times w}$ and $F^{R}\in {\mathbb{R}}^{\frac{c}{2}\times h\times w}$ are concatenated, followed by BatchNorm and GELU operations. The obtained features can be shown as in Equation 4:


(4) $$F^{G}=\sigma_{r}\left(\text{B}\left(F^{L}∥F^{R}\right)\right)$$

where ∥ denotes concatenation along the channel dimension. The processed feature map $F^{G}\in {\mathbb{R}}^{c\times h\times w}$ is then input into the SE block.


(5) $$F^{S}=F^{G}\times \text{s}\left({\text{W}}_{2}\times SiLU\left({\text{W}}_{1}\times Pool\left(F^{G}\right)\right)\right)$$

where s(·) represents the sigmoid activation function, Pool denotes global average pooling operation. Equation 5 can be automatically backpropagated for training, adjusting the values of $W_{1}$ and $W_{2}$ ​ through gradient descent to optimize the model’s performance. Finally, the output feature map $F^{S}\in {\mathbb{R}}^{c\times h\times w}$ is added to our input feature map, resulting in the final representation. The obtained features can be shown as in Equation 6:


(6) $$F^{S}=F^{G}\times \text{s}\left({\text{W}}_{2}\times SiLU\left({\text{W}}_{1}\times Pool\left(F^{G}\right)\right)\right)$$

where ⊕ represents element-wise addition. Subsequently, $F^{D}\in {\mathbb{R}}^{c\times h\times w}$ serves as the input for the next stage and is also the output of the entire D^2^RSE module.

### 3.6 CBAM

**Figure 5:**
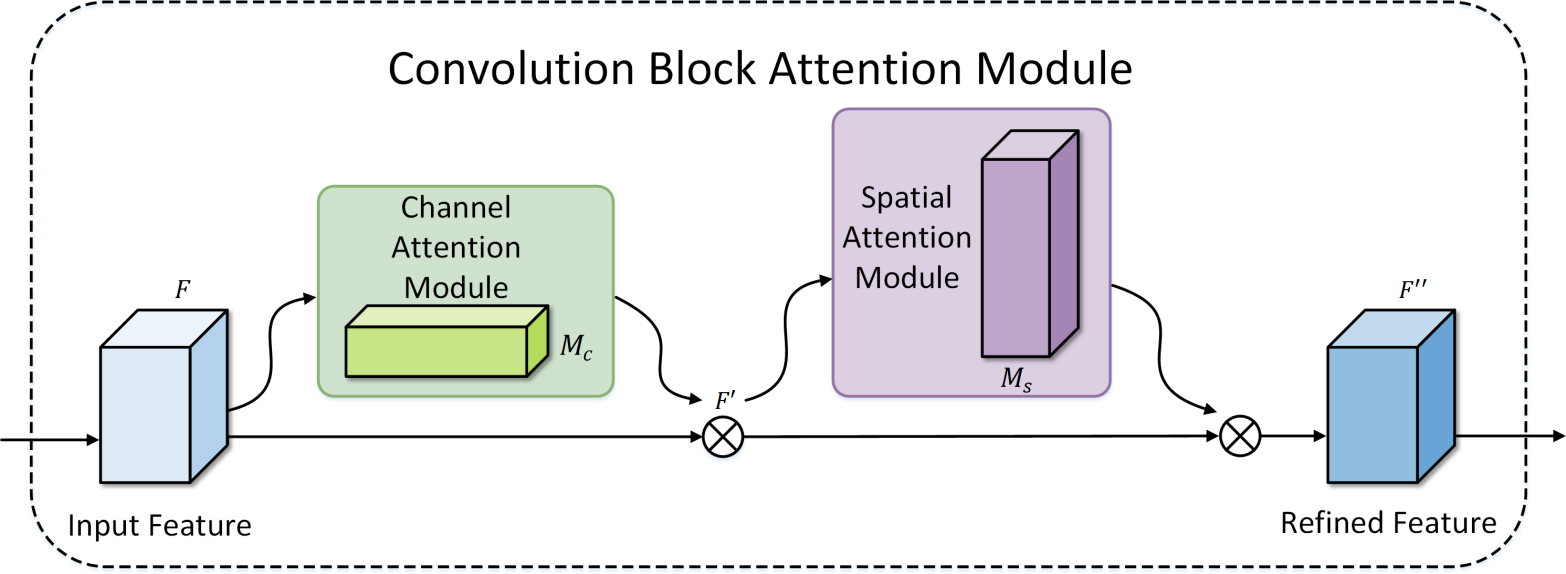
Convolution Block Attention Module.

CBAM introduces spatial and channel attention mechanisms, enhancing the performance of convolutional neural networks. The spatial attention dynamically adjusts the importance of different image positions, while the channel attention adaptively adjusts the importance of different channels. This attention mechanism facilitates more effective capture of critical features in Mpox images, thereby improving performance across various visual tasks. CBAM, proposed by Woo et al. (Woo et al., 2018), is a simple yet effective feedforward convolutional neural network attention module. It infers attention maps in a sequential manner for channel and spatial dimensions independently and then multiplies this map by the input feature map to achieve adaptive feature refinement. Moreover, as CBAM is a lightweight and general-purpose module, it can be seamlessly integrated into any CNN architecture, allowing for end-to-end training alongside the base CNN. **Figure 5** illustrates the structure of CBAM, which includes both channel attention and spatial attention modules.

As illustrated in **Figure 5**, the feature map initially undergoes the channel attention module. This module aggregates spatial information of the feature map using average pooling and max pooling operations, generating two distinct spatial context features, namely $F_{avg}^{c}$ and $F_{max}^{c}$ . Subsequently, these two features are passed through a shared network consisting of a multilayer perceptron (MLP) and a hidden layer to generate the channel attention map $M^{C}\in {\mathbb{R}}^{C\times 1\times 1}$ . Finally, the channel attention map is fused with the original feature map through element-wise summation, forming the ultimate feature output. The feature output is then forwarded to the spatial attention module, which employs two pooling operations to aggregate the channel information of the feature map, generating two 2D maps, $F_{avg}^{S}$ and $F_{max}^{S}$ . These maps are then concatenated and convolved through a standard convolutional layer to produce the final 2D spatial attention map. The mathematical expressions for the aforementioned operations are given by Equations 7, 8.


(7) $$M^{C}=\text{s}\left({\text{W}}_{1}\left({\text{W}}_{0}\left(F_{avg}^{c}\right)\right)+{\text{W}}_{1}\left({\text{W}}_{0}\left(F_{max}^{c}\right)\right)\right)$$

(8) $$M^{S}=\text{s}\left({\text{W}}_{7\times 7}\left(F_{avg}^{S};F_{max}^{S}\right)\right)$$

**Figure 6:**
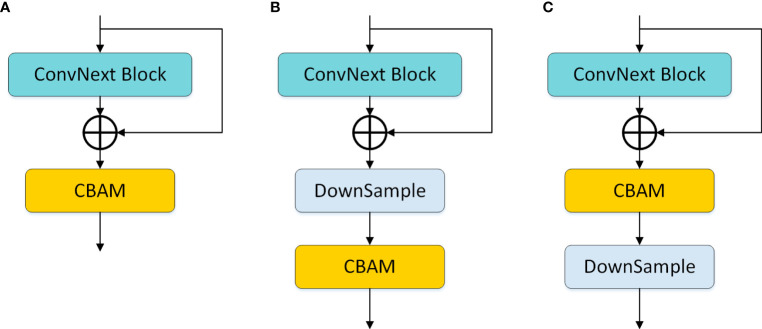
CBAM embedding position design. **(A)** denotes the use of CBAM after each ConvNext block operation; **(B)** denotes the use of CBAM after each downsampling in the model; **(C)** denotes the use of CBAM before each downsampling in the model.

In the study, we further explored the embedding positions of CBAM in the model and designed three variations, as shown in **Figure 6**: (a) indicates the usage of CBAM after each ConvNext block operation; (b) represents the model using CBAM after each downsampling; (c) denotes the model incorporating CBAM before each downsampling. Experimental results revealed that (c) exhibited superior performance, and consequently, our model adopted the design of embedding CBAM after each downsampling.

### 3.7 MpoxNet

**Figure 7:**
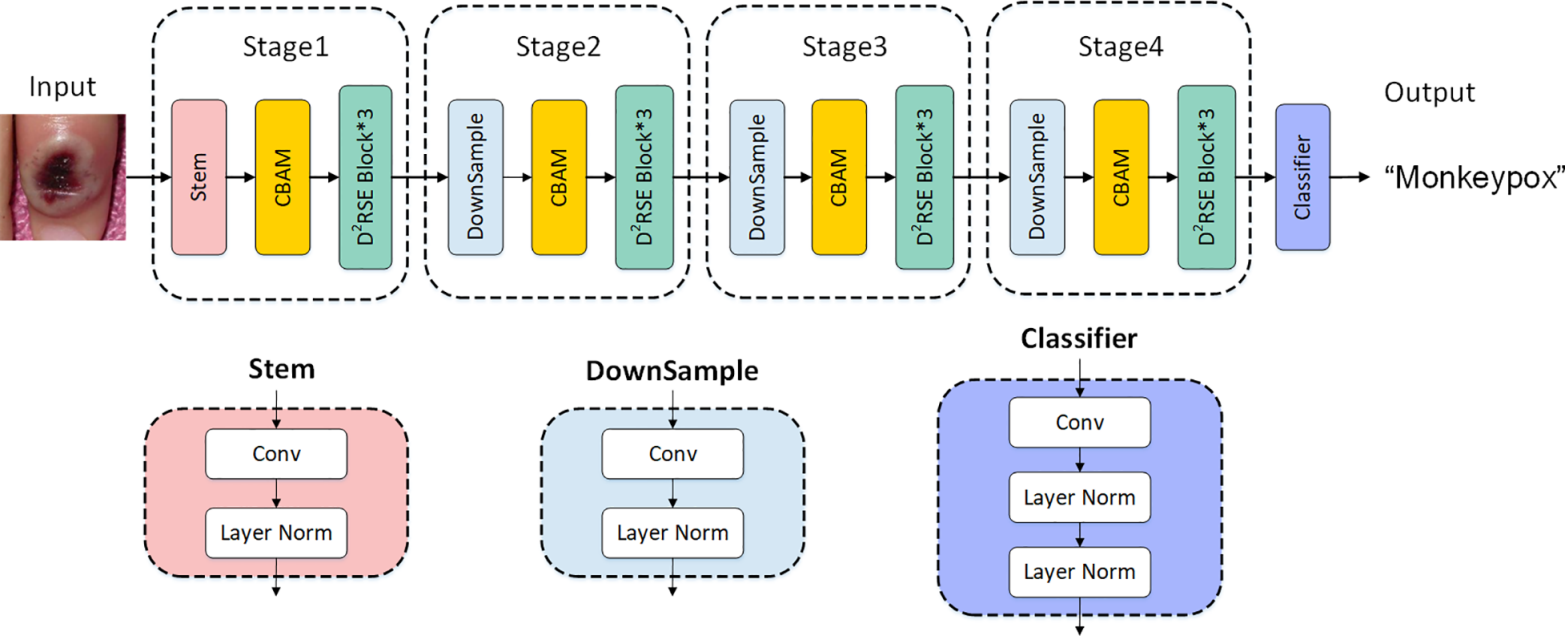
Explanation of MpoxNet network architecture.

Firstly, we devised a dual-branch structure based on the ConvNext Block, named the D2RSE module. Subsequently, we integrated the CBAM module into ConvNext, ultimately constructing a high-precision and lightweight network specifically designed for Mpox classification—MpoxNet. Experimental results demonstrated that MpoxNet excelled in the Mpox classification task. The overall network architecture is illustrated in **Figure 7**.

## 4 Performance evaluation and experimental results

### 4.1 Parameters and evaluation metrics

All experiments were conducted on a high-performance deep learning server with the following hardware configuration: Intel Xeon Silver 4210 CPU with a clock speed of 2.20GHz, NVIDIA GeForce RTX 2080 Ti graphics processing unit with 11GB of video memory, and 128GB of RAM. The deep learning framework employed was Python 3.8.10, Cuda 10.2, torch 1.8.1, and torchvision 0.9.1. The operating system used was Windows 10. During the experiments, consistent training parameters and configurations were applied to train various models. The size of training images was fixed at 224×224, and the batch size was set to 16. Model training utilized the cross-entropy loss function and the AdamW optimizer. In the initial training stage, a warm-up of 1 epoch was performed. The warm-up phase involved gradually updating the learning rate for each iteration using one-dimensional linear interpolation. Following the warm-up, a cosine annealing function was employed to decay the learning rate, starting with an initial learning rate of 0.0005. To ensure fair performance comparisons across different models, no transfer learning was utilized, and each model underwent training for 300 epochs.

Confusion matrix is utilized to calculate performance metrics such as accuracy, precision, recall, and F1 score by comparing predicted labels with actual labels. We employed five widely used performance metrics, including accuracy, precision, recall, and F1-score.

Accuracy is the most commonly used metric, representing the proportion of correctly classified samples out of the total number of samples. The expression is as in Equation 9.


(9) $$Accuracy=\frac{TP+TN}{TP+FN+FP+TN}$$

Precision represents the proportion of true positive samples among those classified as positive. In this context, it is defined as the ratio of samples correctly predicted as Mpox by the model to all samples predicted as Mpox by the model. The expression is as in Equation 10:


(10) $$Precision=\frac{TP}{TP+FP}$$

Recall, also known as sensitivity or true positive rate, measures the proportion of correctly predicted positive samples among all actual positive samples. The recall formula is as shown in Equation 11.


(11) $$Recall=\frac{TP}{TP+FN}$$

The F1-score, also known as the F-measure, is a metric for classification problems that considers both precision and recall. It is the harmonic mean of precision and recall, providing a balance between the two metrics. The F1-score formula is as shown in Equation 12.


(12) $$\text{F}1-score=\frac{2\times Precision\times Recall}{Precision+Recall}$$

TP (True Positive): Instances where the actual class is Mpox, and the model correctly predicts it as Mpox.

TN (True Negative): Instances where both the actual class and the predicted class are not Mpox.

FP (False Positive): Instances where the actual class is not Mpox, but the model incorrectly predicts it as Mpox.

FN (False Negative): Instances where the actual class is Mpox, but the model incorrectly predicts it as not Mpox.

### 4.2 Basic network results

**Figure 8:**
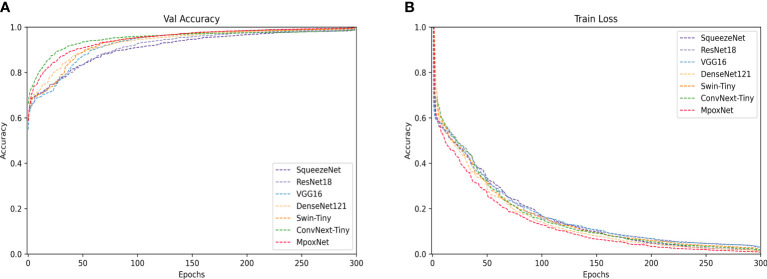
Training and validation of each model on the MSID dataset. **(A)** Trend of the validation accuracy curve of the model with the number of epochs. **(B)** Trend of the training loss curve of the model with the number of epochs.

**Table 2** presents the performance evaluation of our proposed model compared to other trained models. The evaluation results demonstrate that MpoxNet outperforms other networks significantly in terms of prediction accuracy and parameter efficiency on the MSID dataset, achieving an accuracy of 95.28%, precision of 96.40%, recall of 93.00%, and F1-score of 95.80%. Compared to the ConvNext-Tiny model, this paper not only improves the model’s accuracy but also significantly reduces FLOPS and parameter count, being only 30.68% and 31.87% of ConvNext-Tiny, respectively. ConvNext exhibits good fitting performance with an accuracy of 94.33%, and MpoxNet also demonstrates excellent fitting capabilities. The SqueezeNet model has the lowest accuracy at 75.94%, and due to its concise network architecture, the FLOPS and parameter count of this model are only 23.44G and 0.73M, making it unsuitable for practical applications directly. Additionally, com-pared to ResNet18 and ResNet50, ResNet34 performs relatively poorly, with a classification accuracy of only 81.76%. ResNet50 and ResNet18 exhibit similar performance, but ResNet18 has only 44.13% and 47.51% of the FLOPS and model parameters of ResNet50, respectively. Although VGG16 performs well on the test set, its FLOPS and model parameter count are the highest. **Figure 8** illustrates the learning curves of each model during training, including validation accuracy and training loss. In **Figure 8A**, the horizontal axis represents the number of training epochs, and the vertical axis shows the corresponding validation accuracy. In **Figure 8B**, the horizontal axis similarly represents the number of training epochs, and the vertical axis displays the change in training loss. **Figure 8A** indicates that all models begin to converge around 30 epochs and gradually stabilize, reaching complete convergence by 300 epochs. Among these models, MpoxNet achieves the highest validation accuracy at 95.28%. **Figure 8B** reveals relatively consistent trends in the training loss curves for CNN architecture models, with MpoxNet demonstrating out-standing fitting capabilities. In contrast, VGG16 exhibits lower convergence, possibly due to its larger FLOPS and model parameter count.

**Figure 9:**
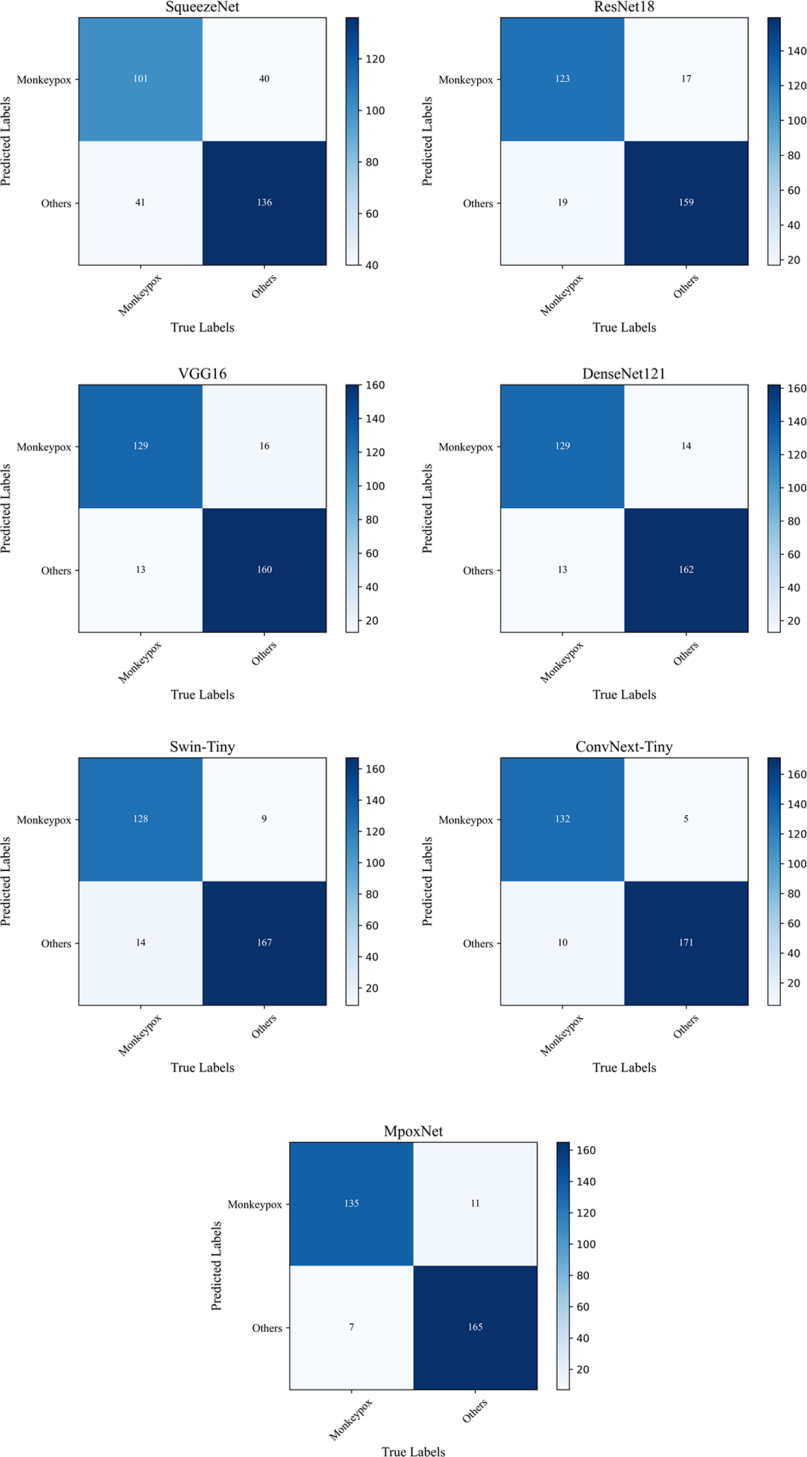
Confusion matrix for different networks.

**Figure 9** displays the confusion matrices for all models tested on the MSID dataset. The confusion matrix is a crucial tool for evaluating the performance of classification problems, and its size depends on the output dimensions. In this binary classification problem, the matrix size is 2×2. The confusion matrix compares the target output and the actual predicted output of the classification model, providing a more intuitive way to identify the strengths and weaknesses of the model and offering detailed performance in-sights. The analysis of the confusion matrix leads to the conclusion that most predictions of the model are accurate. The horizontal axis represents the true labels, while the vertical axis represents the model’s predicted results. The elements on the diagonal indicate the model’s correct predictions, while other elements represent instances of model mispredictions. Nevertheless, due to the high similarity of many images, potential inaccuracies still exist.

### 4.3 CBAM embedding position experiment

In Section 3.5, we proposed three different CBAM embedding position schemes, and the corresponding experimental results are detailed in **Table 3**. Observation reveals that ConvNext, as an advanced classification network, demonstrates robust performance, and the choice of CBAM embedding position in scheme (c) further enhances the model’s performance. Therefore, after each downsampling operation in the network, we opt for the adoption of CBAM.

**Table 3:** Comparison of different CBAM embedding position.

Method	Accuracy(%)	Precision(%)	Recall(%)	F1-score(%)
ConvNext-Tiny	94.33	92.50	91.70	94.80
ConvNext with(a)	94.52	92.63	**94.40**	94.60
ConvNext with(b)	94.45	92.64	93.00	94.10
ConvNext with(c)	**94.65**	**94.30**	93.70	**95.20**

The best results are highlighted in bold text.

### 4.4 Ablation experiments

We validated the effectiveness of the D2RSE and CBAM module in MpoxNet through ablation experiments on the test set, and the specific results are presented in **Table 4**. Observing the experimental data, the CBAM module improved the classification accuracy by 0.32% with almost no increase in the number of model parameters. Combined with the use of the D2RSE module, MpoxNet achieved a classification accuracy of 95.28%, while significantly reducing the FLOPS and parameter count by approximately 69.31% and 68.13%, respectively. This demonstrates the effectiveness of these two modules in enhancing the recognition performance of MpoxNet for Mpox and achieving lightweight model design.

**Table 4:** Comparison of ablation experiments.

D^2^RSE	CBAM	Accuracy(%)	Precision(%)	Recall(%)	F1-score(%)	Flops(G)	Params(M)
–	–	94.33	92.50	95.10	94.80	142.55	27.80
√	–	95.33	94.70	94.80	95.80	**43.71**	**8.83**
–	√	94.65	94.30	93.70	95.20	142.65	27.90
√	√	**95.28**	**96.40**	**93.00**	**95.80**	43.74	8.86

The best results are highlighted in bold text.

### 4.5 Visual interpretation of the model

**Figure 10:**
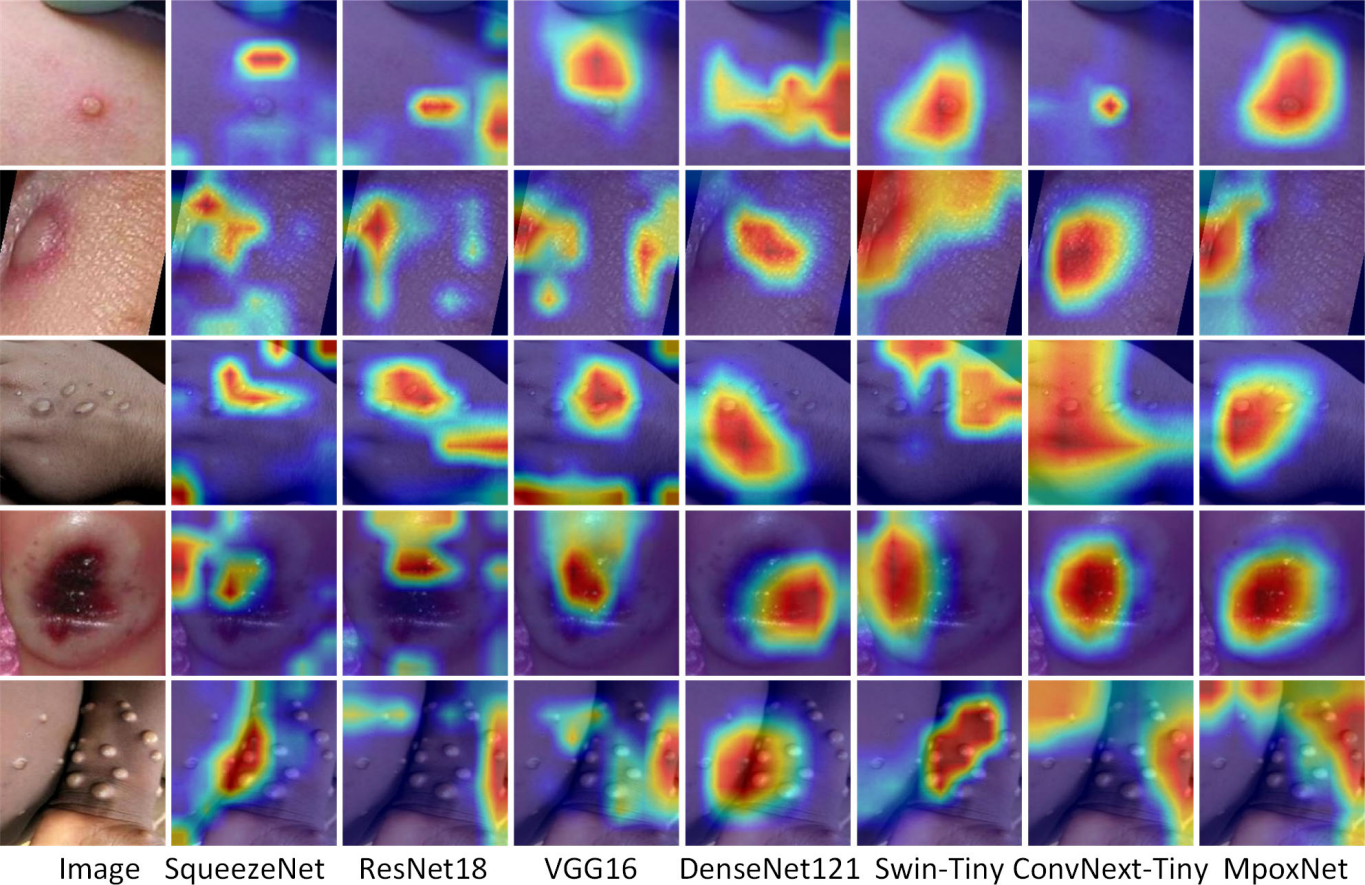
Comparison of heat maps generated by Grad-CAM for each model.

To provide a more intuitive analysis of the Mpox classification process, this study introduced the Gradient-weighted Class Activation Mapping (Grad-CAM) method (Woo et al., 2018). This method generates heatmaps by weighting the model’s output with the gradients of a specified class, highlighting image regions that significantly influence classification decisions. In the heatmap, regions with higher weights are displayed in deeper red, emphasizing their greater impact on the model’s category discrimination. In contrast, regions with lower weights appear in lighter blue, suggesting a milder influence of the image information in these areas on the classification recognition model. **Figure 10** displays the heatmaps generated by Grad-CAM for each model in Mpox classification.

Through the heatmaps, we can clearly observe the core regions that the models focus on. The heatmaps of the SqueezeNet and ResNet18 models exhibit similar features, concentrating on broader areas, with an issue of inaccurate focus on the infected regions. This suggests that they seem to show a considerable interest in widely distributed areas, which may explain their relatively poorer performance in classification. VGG16 and DenseNet121 demonstrate relatively good precision in locating Mpox infection regions. However, their focus areas seem slightly insufficient, indicating potential for further improvement. Swin-Tiny employs a strategy of layer-wise splitting to obtain multi-scale features. Efficient feature communication is achieved through cross-attention networks for these multi-scale features. However, due to its focus extending to the most extensive areas, there is an issue of imprecise attention to Mpox infection regions. Compared to other networks, ConvNeXt-Tiny exhibits a more precise ability to focus on Mpox infection regions, resulting in superior classification outcomes. In MpoxNet, the introduction of the D2RSE module and CBAM module further optimizes the model’s attention to the Mpox region. Therefore, these improvements significantly enhance the classification performance.

## 5 Discussions

In **Table 1**, despite slight variations in experimental parameters and training datasets compared to other methods, MpoxNet attains the highest recognition accuracy. Meanwhile, **Table 2** illustrates that MpoxNet outperforms other networks with the lowest Flops and model parameter count, registering at 23.44G and 0.73M respectively. This allows the MpoxNet to not only serve as a real-time assessment tool but also be easily applied on smartphones for real-time identification and prediction of monkeypox cases. While MpoxNet demonstrates excellent performance in identifying monkeypox lesions, its precision in localizing widely scattered cases of monkeypox is somewhat lacking. In **Figure 10**, although the heatmap can encompass the entire infected area, it fails to accurately mark the positions of individual infection points, highlighting the need for further improvement in this aspect.

## 6 Conclusions

This paper proposes a high-precision lightweight classification network, MpoxNet, based on ConvNext, for the diagnostic classification of Mpox. Firstly, a dual-branch depth separable convolution residual Squeeze and Excitation (D2RSE) module is designed. Then, CBAM is introduced to improve diagnostic accuracy and significantly reduce the model’s parameter count. Our proposed model is compared with SqueezeNet, ResNet18, ResNet34, ResNet50, VGG16, DenseNet121, Swin-Tiny, and ConvNext-Tiny. The proposed model achieves the highest accuracy, precision, recall, and F1-score among all tested models. In ablation experiments, the effectiveness of the D2RSE and CBAM modules is individually verified. By comparing MpoxNet with baseline networks from related papers using the BUSI dataset, the results clearly demonstrate the significant advantages of MpoxNet in terms of performance. This series of comparative experiments validates the crucial roles of the D2RSE and CBAM modules, providing solid evidence for the performance superiority of MpoxNet.

If the results of this study can be implemented, healthcare professionals may be able to improve patient prognosis and reduce medical costs. The research on deep learning and transfer learning techniques for automated Mpox detection not only provides innovative approaches for disease diagnosis but also opens new avenues for ad-dressing diagnostic challenges of other infectious diseases. Breakthroughs in this field will profoundly impact the medical domain, paving the way for the development of future diagnostic tools and methods.

## Data availability statement

The original contributions presented in the study are included in the article/supplementary material. Further inquiries can be directed to the corresponding author.

## Ethics statement

The manuscript presents research on animals that do not require ethical approval for their study. Written informed consent was obtained from the minor(s)’ legal guardian/next of kin for the publication of any potentially identifiable images or data included in this article.

## Author contributions

JS: Software, Visualization, Writing – original draft, Writing – review & editing. BY: Methodology, Supervision, Writing – review & editing. ZS: Validation, Writing – review & editing. JZ: Visualization, Writing – review & editing. YD: Resources, Writing – review & editing. YG: Data curation, Writing – review & editing. YC: Data curation, Writing – review & editing.
